# Rapid Progression of Heterotopic Ossification in Severe Variant of Fibrodysplasia Ossificans Progressiva with p.Arg258Gly in ACVR1: A Case Report and Review of Clinical Phenotypes

**DOI:** 10.1155/2022/5021758

**Published:** 2022-08-25

**Authors:** Kosei Hasegawa, Hiroyuki Tanaka, Natsuko Futagawa, Hiroyuki Miyahara, Hirokazu Tsukahara

**Affiliations:** ^1^Department of Pediatrics, Okayama University Hospital, 2-5-1 Shikata-Cho, Kita-Ku, Okayama 700-8558, Japan; ^2^Department of Pediatrics, Okayama Saiseikai General Hospital, 1-7-18 Ifuku-Cho, Kita-Ku, Okayama 700-8511, Japan; ^3^Department of Pediatrics, Okayama University Graduate School of Medicine, Dentistry and Pharmaceutical Sciences, 2-5-1 Shikata-Cho, Kita-Ku, Okayama 700-8558, Japan

## Abstract

Fibrodysplasia ossificans progressiva (FOP) is a rare skeletal disorder characterized by congenital malformation of the great toes and progressive heterotopic ossification. Malformation of the great toes appears at birth, while heterotopic ossification generally occurs during childhood and rarely occurs during infancy. Classical FOP results from the heterozygous p.Arg206His variant of the ACVR1 gene, which encodes Activin A receptor type 1. Recently, some atypical FOP patients with other ACVR1 gene variants and clinical features that are not observed in classical FOP patients have been reported. Herein, we describe a girl with severe FOP and multiple anomalies, including syndactyly of the hands and feet, nail agenesis, mandibular hypoplasia, heterotopic ossification occurring from infancy, and congenital cardiac malformation. In our patient, we identified de novo occurrence of the heterozygous p.Arg258Gly variant of ACVR1, which has previously been reported in only two severe FOP patients. Heterotopic ossification occurred earlier and more frequently compared with classical FOP patients. We present the time-series changes in heterotopic ossification in our patient and compare her clinical features with those of the previously reported patients with p.Arg258Gly. Our report deepens understanding of the clinical features in severe FOP with p.Arg258Gly and of FOP as a systemic disorder.

## 1. Introduction

Fibrodysplasia ossificans progressiva (FOP; OMIM #135100) is characterized by congenital malformation of the great toes and heterotopic ossification [1]. Additional clinical features of FOP include joint dysplasia, osteochondroma formation, growth plate dysplasia, and cervical vertebrae fusion [1].

FOP is caused by pathogenic variants of the ACVR1 gene, which encodes Activin A receptor type 1 [2]. The heterozygous p.Arg206His variant of ACVR1 located in the glycine/serine-rich domain (GS domain) is typically found in classical FOP patients [2].

ACVR1 is one of the receptors for bone morphogenetic proteins (BMPs). The p.Arg206His variant causes constitutive activation of the basal and ligand-dependent canonical BMP signaling pathway and enhances the potential for osteogenic/chondrogenic differentiation [3]. In addition to BMPs as the main ligands for ACVR1, increased sensitivity to Activin A, a ligand in the TGF*β* pathway, was proposed as one of the causes of FOP [4], and anti-Activin A antibodies are now focused upon as a novel therapeutic strategy for FOP [5].

In classical FOP patients with p.Arg206His, heterotopic ossification generally begins during childhood and rarely occurs during infancy. It is often preceded by a “flare-up,” presenting as painful inflammatory soft tissue swelling. Such flare-ups can be induced by trauma, surgery, intramuscular immunization, mandibular opening for dental work, and flu-like infection, but the precise mechanism remains unknown [1].

Recently, some atypical FOP cases associated with genomic variants other than p.Arg206His have been documented [6]. In atypical FOP cases, heterotopic ossification can occur at any time after childhood or adolescence and can affect not only the great toes but also other toes as well as fingers. Herein, we describe a girl with atypical FOP caused by the heterozygous p.Arg258Gly ACVR1 variant, which has previously been reported in only two severe FOP patients [7]. She exhibited syndactyly of the hands and feet, nail agenesis, and patent ductus arteriosus (PDA) at birth. Heterotopic ossification was first observed at 9 months of age and occurred frequently thereafter. We present the time-series changes in heterotopic ossification in our patient and compare her clinical manifestations with those of the previously reported patients with p.Arg258Gly.

## 2. Clinical Data and Medical History

**Figure 1:**
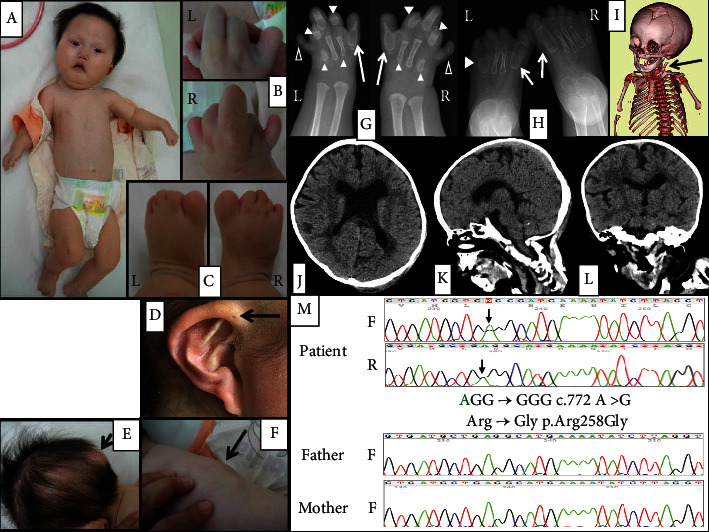
Clinical features, radiological examination findings, and genetic analysis results of our patient. (a–f) Clinical features of our patient at the first visit to our hospital. Left (L); right (R). (a) General appearance. Thin eyebrows, low-set ears, hypoplastic mandible, narrow chest, and shallow umbilicus were observed. (b) Hands. Incomplete syndactyly was observed in both hands. All fingers lacked nails. (c) Feet. Incomplete syndactyly was observed in both feet. All toes lacked nails. (d) Right ear. The lower part of the ear was hypoplastic. A periauricular sinus (arrow) was observed. (e) Flare-up observed at the occipital region of her head (arrow). The hair was sparse. (f) Flare-up observed at her back (arrow). (g–l) Results of radiological examinations. Left (L); right (R). (g) Roentgenograph of the hands at 7 months of age. Agenesis of all distal phalanges, second and fourth middle phalanges, and fifth metacarpal bone, fusion of enlarged first middle metacarpal bone and proximal phalange (arrows), shortened second and fourth metacarpal bones, and proximal phalanges (closed arrowheads), and hypoplasia of the fifth middle phalanges and metacarpal bones (open arrowheads) were observed. Osteochondromas were observed in both first fingers. (h) Roentgenograph of the feet at 2 years of age. Thick first metacarpal bones (arrows) and fused left fourth and fifth metacarpal bones were observed (closed arrowheads). (i) 3D CT analysis at 7 months of age. The left sternocleidomastoid muscle was ossified (arrow). Craniosynostosis was not observed. (j–l) Head CT analysis at 6 years of age. (j) Horizontal view. The septum pellucidum was lacking. The lateral ventricle was enlarged. (k) Sagittal view. The corpus callosum was thin, and a deformed brain stem with bulging dorsal pons was observed. (l) Coronal view. Not only the lateral ventricle but also the third ventricle was enlarged. The sylvian fissure was wide. (m) Results of the genetic analysis of the patient and her parents. A heterozygous base substitution from adenine to guanine at position 772, resulting in an amino acid change from arginine to glycine at 258, was observed in the patient (arrow). This substitution was not observed in either of her parents. Forward strand (F); reverse strand (R).

A 9-month-old Japanese girl was referred to our hospital because of multiple congenital anomalies (Figures 1(a)–1(d)) and recurrent tumorous lesions in her occipital region and back that had occurred from 7 months of age (Figures 1(e) and 1(f)).

She was born at 39 weeks of gestational age from non-consanguineous parents. Her parents had no past history of congenital anomalies or heterotopic ossification. Her birth weight was 2804 g and asphyxia was not observed. Her multiple congenital anomalies included soft tissue syndactyly of the hands and feet without nails, low-set ears, auricular anomaly, and hypoplastic mandible (Figures 1(a)–1(d)). PDA was identified after birth and closed by a catheter device at 1 year 6 months of age. Her chromosomal karyotype was 46, XX.

At the first visit to our hospital, small malformed teeth, thin eyebrows, shallow umbilicus (Figure 1(a)), and sparse hair (Figure 1(e)) were observed in addition to the anomalies found at birth. Radiologic analysis revealed multiple symmetrical abnormalities of the phalanges and metacarpal bones in the hands and feet and heterotopic ossification of the sternocleidomastoid muscle (Figures 1(g)–1(i)). Head CT analysis at 6 years of age revealed enlargement of the lateral and third ventricles, which was not progressive, lack of septum pellucidum, hypoplastic corpus callosum, and deformed brain stem including bulging dorsal pons (Figures 1(j)–1(l)).

From the heterotopic ossification and abnormalities of the great toes, we suspected FOP. After informed consent was obtained from her parents, a genetic analysis of the ACVR1 gene was conducted. The genetic analysis was approved by the Ethical Committee of Okayama University Hospital (2016.6.29:1606-021). All the procedures in the study were performed in accordance with the 1964 Helsinki Declaration and 2003 Japanese Ethical Guidelines for Clinical Research, as well as their later amendments. The sequence reads were aligned with reference sequences from GenBank (NM_001105.4). As a result, a heterozygous single base substitution, c.772 A > G, in the coding region of ACVR1, resulting in p.Arg258Gly, was identified in our patient, while other known pathogenic variants, including p.Arg206His, were absent (Figure 1(m)). The identified substitution was not present in either of her parents, indicating that the variant had occurred de novo. The p.Arg258Gly variant was not found in SNP databases, including the dbSNP (https://www.ncbi.nlm.nih.gov/snp/) and Genome Aggregation Database (https://gnomad.broadinstitute.org). The underlying variant for p.Arg258Gly was located in the kinase domain of ACVR1. Upon analysis with three online software programs, SIFT (https://provean.jcvi.org/index.php), PolyPhen-2 (https://genetics.bwh.harvard.edu/pph2/), and MutationTaster (https://www.mutationtaster.org), p.Arg258Gly was predicted to be a disease-causing or damaging variant, and p.Arg258 was found to be maintained in various species. The CADD (https://cadd.gs.washington.edu) score for this substitution was 29.5. The variant was previously reported in two patients with severe FOP and multi-system involvement [7]. Taken together, the p.Arg258Gly variant is considered “pathogenic (PS1, PS2, PP3, and PP4)” based on the ACMG criteria [8]. The data for the variant were deposited to the Global Variome shared LOVD (https://databases.lovd.nl/shared/variants/0000828134#00002047).

**Figure 2:**
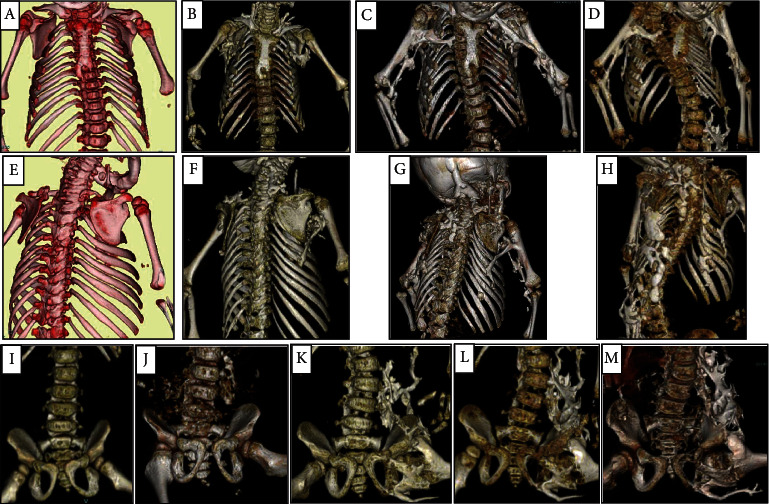
Time-course series of heterotopic ossification (HO). (a–d) Front chest and upper limbs. (e–h) Back chest. (a, e) At 10 months of age, HO was not observed. (e) Cervical vertebrae fusion was observed. (b, f) At 26 months of age, HO was observed from the bilateral axillary and humerus. (c, g) At 42 months, HO was observed between the humerus and radius and between the humerus and thoracic cage. (d, h) Scoliosis due to multiple HO occurrences was observed at 106 months. (i–m) Lumbar spine and pelvis. (i) At 26 months of age, HO was not observed. (j) At 42 months of age, HO was observed from the left transverse process of the lumbar spine. (k) At 78 months of age, HO was observed from the neck of the left femur and connected the left transverse process and left femur thereafter. (l) At 106 months of age. (m) At 121 months of age.

After the genetic diagnosis of FOP, oral prednisolone was administered when a flare-up occurred. However, heterotopic ossification following the frequent flare-ups was not suppressed (Figure 2). The patient is currently 12 years of age and is healthy. However, her hair has been almost completely lost except for the forehead region, and her limbs except for the right leg cannot be moved due to joint contracture caused by the broad heterotopic ossification.

## 3. Discussion

The skeletal abnormalities found in patients with p.Arg258Gly ACVR1 were atypical and more severe than those reported in classical FOP patients and other atypical FOP patients in the following points: (1) heterotopic ossification occurred at an earlier age and was more frequent and (2) the feet and hands were both affected, presenting with short fingers and toes and lack of nails [7]. Through ACVR1, BMP signaling contributes to not only ossification but also large and small joint formation [1, 9]. In FOP with pathogenic ACVR1 variants, abnormally increased BMP signaling causes heterotopic ossification, joint dysplasia, growth plate dysplasia, and osteochondroma formation. The severe clinical phenotypes found in patients with p.Arg258Gly suggest that this variant causes the strongest exacerbation in BMP signaling among the pathogenic variants found in classical and atypical FOP patients. The pathogenic ACVR1 variants p.Arg206His and p.Arg258Gly found in FOP patients were also identified in tissues from patients with diffuse pontine glioma (DPG), a lethal pediatric brain tumor [10]. However, the degree of SMAD1/5 phosphorylation in tissues with p.Arg258Gly varied according to the experimental conditions: stronger than or similar to other pathogenic variants found in FOP patients and DPG patients with p.Arg206His, with or without a ligand (BMPs or Activin A), and cell type-dependent [4, 10]. In ACVR1, p.Arg258 is a highly conserved amino acid. p.Arg258Ser [6] and p.Arg258Trp [11] also cause FOP, but the clinical phenotypes are milder than that of p.Arg258Gly. Responses to ligands such as BMPs and Activin A in not only the canonical BMP pathway but also the non-canonical BMP pathway may be involved in the different phenotypes among the ACVR1 variants.

Brain anomalies are one of the clinical manifestations in FOP patients. Our patient had a brainstem anomaly and non-progressive ventricular enlargement, as reported previously [12], but also lacked the septum pellucidum and had a hypoplastic corpus callosum. Hypoplasia of the corpus callosum was also found in one of the reported patients with p.Arg258Gly (Table 1). Spatiotemporal control of BMP signaling is important for brain development [13]. Therefore, dysregulated BMP signaling may lead to the lack of septum pellucidum and hypoplastic corpus callosum, although the detailed mechanisms and relationships remain to be clarified.

BMP signaling is also implicated in development of the heart [14]. Clinically, some variants of ACVR1 were reported to cause congenital heart defects, but not FOP [15]. However, these variants showed loss of function. In classical FOP patients, congenital heart disease has rarely been reported. Specifically, only one classical FOP patient with ventricular septum defects has been described, but the direct relationship with FOP was unknown [16]. Our patient and one of the reported patients with p.Arg258Gly had PDA (Table 1). Before birth, prostaglandin E2 (PGE2) not only causes vasodilation of the ductus arteriosus (DA) but also activates DA intima thickening during mid-to-late pregnancy, leading to the narrowing of the DA vascular lumen [17]. After birth, the decrease in PGE2 causes vasospasms and DA closure. It is unknown whether constitutively activated BMP signaling affects PGE2-EP4 receptor signaling during infancy. However, one of the reported patients with p.Arg258Gly had PDA that required a surgical intervention at 15 days of age. We believe that there may be a direct or indirect relationship between DA closure and abnormally activated BMP signaling during certain fetal periods.

During dental development, BMP signaling forms a signaling network with Wnt, Fgf, Shh, and Eda [18]. However, dental abnormalities are rare clinical symptoms in FOP patients. Nevertheless, all three patients with p.Arg258Gly, comprising our patient and the two reported patients, had small malformed teeth (Table 1). Thus, the dental abnormalities observed in these patients may be specific symptoms caused by p.Arg258Gly.

Diffuse thinning of the hair that occurred in middle age was previously observed in FOP patients [19]. However, it was unclear whether this thinning was a primary feature or a secondary feature to nutritional deficiency caused by lockjaw. BMP signaling has also been implicated in quiescence of hair follicle stem cells (HFSCs) and the hair cycle [20]. Reduced BMP signaling leads to activation of quiescent HFSCs and disruption of the hair cycle. In our patient, thinning of the hair and eyebrows was present from 9 months of age and her hair was almost completely lost at 12 years of age. The other two patients with p.Arg258Gly also exhibited thinning of their hair at an early age (Table 1). Although it remains unknown why thinning of the hair occurs in FOP patients, we consider that increased BMP signaling may abolish the quiescence of HFSCs and accelerate the hair cycle. We also believe that hair thinning and early hair loss are some of the primary clinical symptoms of FOP.

In conclusion, we identified the p.Arg258Gly variant of ACVR1 in a patient with severe atypical FOP. It remains to be clarified how dysregulated BMP signaling caused her atypical and very severe clinical findings. Our results broaden the clinical and radiological features of severe FOP with p.Arg258Gly ACVR1 and provide the important insight that FOP is not only a skeletal disease but also a systemic disease that affects many organs.

## Figures and Tables

**Table 1:** Clinical characteristics of severe FOP patients with the heterozygous p.Arg258Gly variant of ACVR1.

		[7]		Present case
		Patient 1	Patient 2	
Variant/mode of inheritance		Heterozygous c.772G > A; p.Arg258Gly/de novo		
Chromosome		46, XX	46, XY	46, XX
Age at onset of heterotopic ossification		16 months	11 months	9 months
Heterotopic ossification-induced multiple joint contracture		+		
Fusion of cervical vertebra		+		
Four-limb digit reduction anomalies with no nails and soft tissue syndactyly		+		
Dysmorphic facial features	Microretrognathia	+		
	Low-set dysmorphic ears	+		
	Hypertelorism	−	+	−
	Depressed nasal bridge	+	−	+
	Sparse hair	+		
	Small malformed teeth	+		
Brain anomalies	Hypoplasia of brainstem	+	n.a.	+
	Hydrocephalus	+	n.a.	+
	Agenesis/hypoplasia of corpus callosum		+	+
Craniosynostosis		n.a.	+	-
Patent ductus arteriosus		−	+	+
Genital anomalies		−	+	−
Sensorineural hearing loss		+	n.a.	+
Gross motor delay		+		
Other malformations			Left renal duplication	

## Data Availability

The data that support the findings of this study are openly available in Global Variome shared LOVD at https://databases.lovd.nl/shared/variants/0000828134#00002047.

## References

[1] Kaplan F. S., Al Mukaddam M., Stanley A., Towler O. W., Shore E. M. (2020). Fibrodysplasia ossificans progressiva (FOP): a disorder of osteochondrogenesis. *Bone*.

[2] Shore E. M., Xu M., Feldman G. J. (2006). A recurrent mutation in the BMP type I receptor ACVR1 causes inherited and sporadic fibrodysplasia ossificans progressiva. *Nature Genetics*.

[3] Fukuda T., Kohda M., Kanomata K. (2009). Constitutively activated ALK2 and increased SMAD1/5 cooperatively induce bone morphogenetic protein signaling in fibrodysplasia ossificans progressiva. *Journal of Biological Chemistry*.

[4] Hino K., Ikeya M., Horigome K. (2015). Neofunction of ACVR1 in fibrodysplasia ossificans progressiva. *Proceedings of the National Academy of Sciences of the USA*.

[5] Hatsell S. J., Idone V., Wolken D. M. (2015). ACVR1R206H receptor mutation causes fibrodysplasia ossificans progressiva by imparting responsiveness to activin A. *Science Translational Medicine*.

[6] de Brasi D., Orlando F., Gaeta V. (2021). Fibrodysplasia ossificans progressiva: a challenging diagnosis. *Genes*.

[7] Kaplan F. S., Kobori J. A., Orellana C. (2015). Multi-system involvement in a severe variant of fibrodysplasia ossificans progressiva (ACVR1 c.772G>A; R258G): a report of two patients. *American Journal of Medical Genetics*.

[8] Richards S., Aziz N., Bale S. (2015). Standards and guidelines for the interpretation of sequence variants: a joint consensus recommendation of the American college of medical genetics and genomics and the association for molecular pathology. *Genetics in Medicine*.

[9] Towler O. W., Shore E. M. (2022). BMP signaling and skeletal development in fibrodysplasia ossificans progressiva (FOP). *Developmental Dynamics*.

[10] Wu G., Diaz A. K., Paugh B. S. (2014). The genomic landscape of diffuse intrinsic pontine glioma and pediatric non-brainstem high-grade glioma. *Nature Genetics*.

[11] Cappato S., Traberg R., Gintautiene J., Zara F., Bocciardi R. (2021). A case of fibrodysplasia ossificans progressiva associated with a novel variant of the ACVR1 gene. *Molecular Genetics & Genomic Medicine*.

[12] Bertamino M., Severino M., Schiaffino M. C. (2015). New insights into central nervous system involvement in FOP: case report and review of the literature. *American Journal of Medical Genetics*.

[13] Bond A. M., Bhalala O. G., Kessler J. A. (2012). The dynamic role of bone morphogenetic proteins in neural stem cell fate and maturation. *Developmental Neurobiology*.

[14] Feulner L., van Vliet P. P., Puceat M., Andelfinger G. (2022). Endocardial regulation of cardiac development. *Journal of Cardiovascular Development and Disease*.

[15] Smith K. A., Joziasse I. C., Chocron S. (2009). Dominant-negative ALK2 allele associates with congenital heart defects. *Circulation*.

[16] Marseglia L., D’Angelo G., Manti S. (2015). Fibrodysplasia ossificans progressiva in a newborn with cardiac involvement. *Pediatrics International*.

[17] Yokoyama U. (2015). Prostaglandin E-mediated molecular mechanisms driving remodeling of the ductus arteriosus. *Pediatrics International*.

[18] Lan Y., Jia S., Jiang R. (2014). Molecular patterning of the mammalian dentition. *Seminars in Cell & Developmental Biology*.

[19] Morales-Piga A., Bachiller-Corral J., Trujillo-Tiebas M. J. (2012). Fibrodysplasia ossificans progressiva in Spain: epidemiological, clinical, and genetic aspects. *Bone*.

[20] Plikus M. V., Mayer J. A., de la Cruz D. (2008). Cyclic dermal BMP signalling regulates stem cell activation during hair regeneration. *Nature*.

